# Binding of the Lactococcal Drug Dependent Transcriptional Regulator LmrR to Its Ligands and Responsive Promoter Regions

**DOI:** 10.1371/journal.pone.0135467

**Published:** 2015-08-12

**Authors:** Jan Pieter van der Berg, Pramod Kumar Madoori, Amalina Ghaisani Komarudin, Andy-Mark Thunnissen, Arnold J. M. Driessen

**Affiliations:** 1 Molecular Microbiology, Groningen Biomolecular Sciences and Biotechnology Institute, University of Groningen, Groningen, The Netherlands; 2 Department of Biophysical Chemistry, Groningen Biomolecular Sciences and Biotechnology Institute, University of Groningen, Groningen, The Netherlands; Saint Louis University, UNITED STATES

## Abstract

The heterodimeric ABC transporter LmrCD from *Lactococcus lactis* is able to extrude several different toxic compounds from the cell, fulfilling a role in the intrinsic and induced drug resistance. The expression of the *lmrCD* genes is regulated by the multi-drug binding repressor LmrR, which also binds to its own promoter to autoregulate its own expression. Previously, we reported the crystal structure of LmrR in the presence and absence of the drugs Hoechst 33342 and daunomycin. Analysis of the mechanism how drugs control the repressor activity of LmrR is impeded by the fact that these drugs also bind to DNA. Here we identified, using X-ray crystallography and fluorescence, that riboflavin binds into the drug binding cavity of LmrR, adopting a similar binding mode as Hoechst 33342 and daunomycin. Microscale thermophoresis was employed to quantify the binding affinity of LmrR to its responsive promoter regions and to evaluate the cognate site of LmrR in the *lmrCD* promoter region. Riboflavin reduces the binding affinity of LmrR for the promoter regions. Our results support a model wherein drug binding to LmrR relieves the LmrR dependent repression of the *lmrCD* genes.

## Introduction

The emergence of antibiotic resistant bacteria is becoming a serious threat for the present day public healthcare. Several resistance mechanisms contribute to the antimicrobial resistance phenotype of pathogenic bacteria, including enzymatic inactivation, reduced influx and substituted antibiotic targets [1]. The presence of dedicated drug extrusion transport proteins is a major contributor to the multidrug resistance (MDR) phenotype of microbes. Certain bacterial transport proteins have been shown to cause MDR, like AcrAB and MdfA of *Escherichia coli* [2] and NorA of *Staphylococcus aureus* [3].

The bacterium *Lactococcus lactis*, which is widely used in dairy industry, contains a number of multidrug transporters, which have been used as a model to study bacterial multidrug resistance [4]. A major resistance determinant in this bacterium is LmrCD [5,6], which belongs to the ATP-binding cassette (ABC) superfamily and was shown to transport a wide variety of drugs. Likewise, *L*. *lactis* harbors the drug transporters LmrA [7], the first identified bacterial ABC transporter involved in drug resistance, and LmrP [8], a secondary transporter involved in multidrug resistance. LmrCD consists of two ABC half-transporters, LmrC and LmrD, which are homologous (27% sequence identity). Together they form a functional heterodimer. Growth studies of *L*. *lactis* in the presence of increasing concentrations of toxic chemicals, like rhodamine 6G and daunomycin, resulted in an enhanced MDR phenotype, which was shown to be caused by an increased expression of the *lmrCD* gene [6]. A regulatory protein, LmrR, encoded in the DNA region upstream of *lmrCD* was also upregulated under these conditions, although due to a point mutation LmrR was rendered inactive causing a constitutive expression of the *lmrCD* genes.

LmrR belongs to the PadR-like family of transcriptional regulators (Pfam PF03551), which is named after a regulatory protein involved in the regulation of expression of phenolic acid decarboxylase (*pad*) genes [9]. LmrR was shown to be a transcriptional repressor of *lmrCD* expression and an autoregulator of its own expression [10]. Crystal structures of LmrR bound with its ligands Hoechst 33342 (H33342) and daunomycin have been elucidated [11]. LmrR forms a dimer with each monomer comprising a typical winged helix-turn-helix DNA binding domain and a C-terminal helix involved in dimerization. In between the two monomers is a large central pore that serves as the drug-binding site. It was shown that two tryptophan residues (Trp96 and Trp96’, the apostrophe denotes the other subunit in the dimer) in the central pore are essential for the binding of ligands, which are mostly planar heterocyclic compounds. The hydrophobic tryptophan side chains, together with other adjacent hydrophobic residues, stabilize the ligand in the binding site. In depth analysis of the LmrR-DNA binding revealed that LmrR interacts with two specific DNA motifs in the operator region of both *lmrCD* and *lmrR* [12]. The LmrR binding site in the promoter/operator (p/o) region of *lmrCD* is a typical PadR consensus sequence and binds LmrR with high affinity. An incomplete PadR motif is located in the p/o of *lmrR*, this palindromic sequence is only weakly recognized by LmrR. A proposed mechanism for the binding of LmrR to the p/o regions and the regulation of *lmrCD* and *lmrR* is as followed: the binding of two LmrR dimers to P*lmrCD*, together with an extensive binding of P*lmrR* by multiple LmrR dimers, results in a repression of *lmrCD* and a strong autorepression of *lmrR*. The current model is that intracellular presence of a toxic compound at relatively low concentrations will cause LmrR to bind the drug, which in turn will release the LmrR dimer from P*lmrCD*, thus allowing initiation of *lmrCD* expression. At higher drug concentration the multiple LmrR dimers bound to *PlmrR* will also be released and this derepression leads to an increased expression of both *lmrCD* and *lmrR* [12].

A major problem with the drugs that bind to LmrR is that they so far are all DNA-binding drugs, like H33342, daunomycin and ethidium [11], which interact with DNA via groove binding or intercalation. This has precluded validation of the above model using EMSA assays. In this study we identify, using fluorescence and X-ray crystallography, that LmrR binds the vitamin riboflavin (RBF), which has a planar hydrophobic core structure but does not bind to DNA like the other LmrR ligands. We then examined LmrR binding to its p/o regions in the presence and absence of using Microscale Thermophoresis (MST). This new methodology monitors the diffusion of particles in a microscopic temperature gradient, depending on several biochemical properties, like charge, hydration shell and molecular mass, particles will behave differently in the gradient [13]. MST is ideally suited to examine the interaction of DNA binding proteins with their cognate substrate DNA. We characterize the minimal binding region necessary for LmrR interaction including the palindromic PadR motif, and analyzed the effect of ligand binding on the interaction between LmrR and DNA.

## Materials and Methods

### Bacterial strains and growth conditions

*E*. *coli* Bl21 (DE3)C43 containing the pET17b_LmrR_strep plasmid [14] was grown in Luria Bertani (LB) medium supplemented with 100 μg/ml ampicillin at 37°C.

### Protein overproduction and purification

An overnight culture of *E*. *coli* containing pET17b_LmrR_strep was diluted in fresh LB with ampicillin and grown till an optical density at 600 nm (OD_600_) of 0.8. Subsequently, IPTG was added to a final concentration of 1 mM and cells were grown overnight at 30°C. Cells were collected by centrifugation (6000 rpm, JLA10.500 rotor, 20 min, 4°C, Beckman), and resuspended in 50 mM NaH_2_PO_4_, pH 8.0, 150 mM NaCl, and 10% glycerol and again centrifuged as before. The resulting cell pellet was frozen at -20°C. After thawing, the pellet was resuspended in resuspension buffer (50 mM potassium phosphate buffer, pH 8, 150 mM NaCl, 10% glycerol) and the cells were disrupted twice using a one-shot cell disruptor (Constant Systems, Daventry, UK) at 13 kPsi, with 1 mM PMSF added between the two cell disruption steps to inhibit proteases. Following the cell disruption, MgCl_2_ (final concentration 10 mM) and 1 mg/ml DNaseI was added and the lysate was incubated for 1 hour at 30°C. Using a long needle, the cell lysate was sheered and centrifuged (15000 rpm, SS34 rotor, 20 min, 4°C, Sorvall) to remove the debris, whereupon the cell free extract was filtered through a 45 μm filter. The supernatant was incubated with 6 mL Strep-tag Tactin slurry (50% Strep-tag Tactin in 50 mM potassium phosphate buffer, pH 8, 150 mM NaCl, 10% glycerol), for one hour on a rotary shaker at 4°C. Subsequently, the material was loaded on a gravity flow column, and washed 5 times with two column volumes of resuspension buffer. LmrR-Strep was eluted with 6 column volumes of resuspension buffer containing 2.5 mM desthiobiotin. LmrR-Strep containing fractions were desalted to 50 mM PB, pH 8 using a Econo-PAC 10DG column (Bio-Rad) and applied to a 5 mL Heparin column (pre-equilibrated with 50 mM PB, pH 8), LmrR was eluted using a gradient of 2 M NaCl (0 to 100%, 10 min). Fractions were analyzed on a 12% SDS polyacrylamide gel, stained with Coomassie Brilliant Blue, and the protein concentration and purity was assessed using a UV-VIS spectrometer.

### Crystallization of LmrR•RBF

LmrR was produced as an untagged protein by nisin-induced overexpression in *L*. *lactis* and purified as described earlier [11]. The complex of LmrR with riboflavin was prepared by mixing protein (8 mg/ml final concentration) and ligand (1.5 mM final concentration) in a 1:5 molar ratio in a solution containing 20 mM Tris-HCl, pH 8.0, 280 mM NaCl and 1 mM EDTA. Initial crystallization conditions were obtained by sparse-matrix screening, using the PACT and JCSG+ commercial kits (Molecular Dimensions) and with the help of a Douglas Instruments Oryx-6 crystallization robot. Manual optimization using a sitting-drop vapor diffusion setup resulted in a final crystallization solution, containing 0.1 M Tris-HCl, pH 8.5, 17% PEG 2000 monomethyl ether (MME) and 0.2 M tri-methylamine N-oxide. Crystals grew overnight from drops containing 1 μl reservoir and 1 μl of the protein-drug mixture at 295 K.

### Data collection and structure determination

X-ray diffraction data were collected at 100 K from a single flash-cooled crystal on beamline ID23-2 at the European Synchrotron Radiation Facility (ESRF). The cryo-protecting solution was prepared from the crystallization solution by increasing the PEG 2000 MME to 40% and adding 0.1 M NaCl and 0.2 mM ligand. Data were processed with XDS [15] and scaled, merged, and reduced with programs from the CCP4 suite [http://www.ccp4.ac.uk/]. The structure of LmrR•RBF was solved by molecular replacement with the program PHASER from the CCP4 program suite, using a single subunit of the unliganded LmrR dimer as a search model (PDB entry 3F8B). The model was improved in several cycles, by restrained refinement using the programs Phenix.refine [16] and Refmac5 [17], alternated by manual model building using COOT [18]. Subsequently, the ligand was modeled into excess density observed in the central LmrR pore. Evaluation of the interaction geometries, real-space correlation factors and short molecular dynamics runs was used to guide the docking of the ligand. Molecular dynamics (200 ps) was performed with the Yasara Structure software package [http://www.yasara.org], version 15.3.8, using included macros in combination with the YASARA2 force field. Fixed translation libration screw (TLS) parameters were determined using the TLS motion detection server [19] and then used in the subsequent rounds of structure refinement. In the last stages of the refinements, water molecules were placed and retained in the model by strict criteria of difference density, B-factor cutoffs, and hydrogen-bonding capacity. The quality of the final models was checked using MolProbity [20]. Selected data collection and refinement statistics are presented in Table 1. The coordinates and structure factors have been deposited in the PDB with accession code 4ZZD.

**Table 1:** Relevant crystallographic statistics of LmrR•RBF.

*Data collection*		
Space group		*P*4_3_2_1_2
Cell dimensions	a, b, c (Å)	35.2, 35,2, 179.7
Resolution range*		35–2.35 (2.53–2.35)
No. of unique reflections		4872
Completeness (%)*		98.8 (99.3)
Multiplicity*		3.3 (3.4)
*R*_merge_ *		0.057 (0.37)
*I/σI* *		15.3 (3.2)
Wilson B-factor (Å^2^)		36.4
*Refinement*		
Resolution range		30–2.35
*R*_*factor*_ */R*_*free*_		0.21/0.27
No. of atoms in asymmetric unit,		
average B factor (Å^2^)		
	Protein (chain A)	809, 43.2
	Solvent	17, 32.4
	Ligand	54, 49
Rmsd		
	Bond lengths (Å)	0.01
	Bond angles (°)	1.01
Ramachandran analysis, validation		
	Preferred regions (%)	100
	Allowed regions (%)	0.0
	Outliers (%)	0.0
	Molprobity score	1.5

*Values in parentheses refer to the highest resolution shell

### Fluorescence-based binding assay

LmrR-RBF binding was measured by fluorescence titration using a Fluorimeter (Photon Technology International) at 25°C. To a 3-mL stirred cuvette with 100 nM purified LmrR dimer in binding buffer (20 mM Tris-HCl, pH 8.0, 150 mM NaCl and 1 mM EDTA), RBF (37.5 μM stock in binding buffer) was added in 2 μL steps, which results in an increase of 25 nM per step. RBF fluorescence at 523 nm was measured after every titration step, using an excitation wavelength of 435 nm. As a control, RBF was also titrated to the buffer lacking LmrR. After measurement the fluorescence quenching was calculated from the change between the control and samples containing LmrR. In addition, two independent titrations were carried out with 1.4 μM LmrR dimer in binding buffer, using a 1-ml cuvette and stepwise additions of 5 μL RBF (112 μM stock). After correcting for dilution effects, fluorescence quenching data at the higher protein concentration were obtained by averaging the data of the two measurements. From each set of plotted fluorescence quenching data (at low or high protein concentration) the dissociation constant was calculated by non-linear regression data fitting, using the program SigmaPlot [http://www.sigmaplot.com], with the equation as described in [21].

### LmrR—promoter binding assay

Responsive promoter regions of both the *lmrR* and *lmrCD* genes were amplified using primers listed in Table 2. Forward primers contain a 5’- Cy5 fluorophore modification for use in the thermophoresis assay, modified primers were ordered from Sigma-Aldrich. A serial dilution of purified LmrR-Strep was prepared in Binding buffer (20 mM Tris.HCl, pH 8.0; 1 mM EDTA). To this serial dilution Cy5 –labeled promoter DNA was added to a final concentration of 50 nM and the total mixture was incubated for 30 minutes at 30°C. For ligand binding studies an excess (50 μM) of riboflavin was added. Samples were loaded in standard treated thermophoresis capillaries and protein—DNA binding was measured using a Monolith NT.115 (Nanotemper Technologies), with 30% LED power and 40% laser power unless stated otherwise. From the binding curves the apparent dissociation constant (K_d_) was calculated using the Hill equation.

**Table 2:** Primers used in this study.

Name	5’– 3’ sequence	Reference
P*lmrCD* F	Cy5- CGATTCATTCCTTACTTTAAATTC	This work
P*lmrCD* R	AAGATTGAGAATAAGGCAACCC	(12)
P*lmrCD* R -62bp	TTCTAGAGTTAAATAATGTAAAC	This work
P*lmrCD* R muta	TTCCTTTCTAGAGTTAAATAACCCAAACTAC	This work
P*lmrCD* R noPadR	ACTACTTTACATTAAATTG	This work
P*lmrR* F	CGGAGATGATTTTTTCTTATCTTATATAG	(12)
P*lmrR* R	CTCCTTGTTTTAGGACATTGAGC	(12)

## Results

### Structure determination of riboflavin-bound LmrR

**Fig 1:**
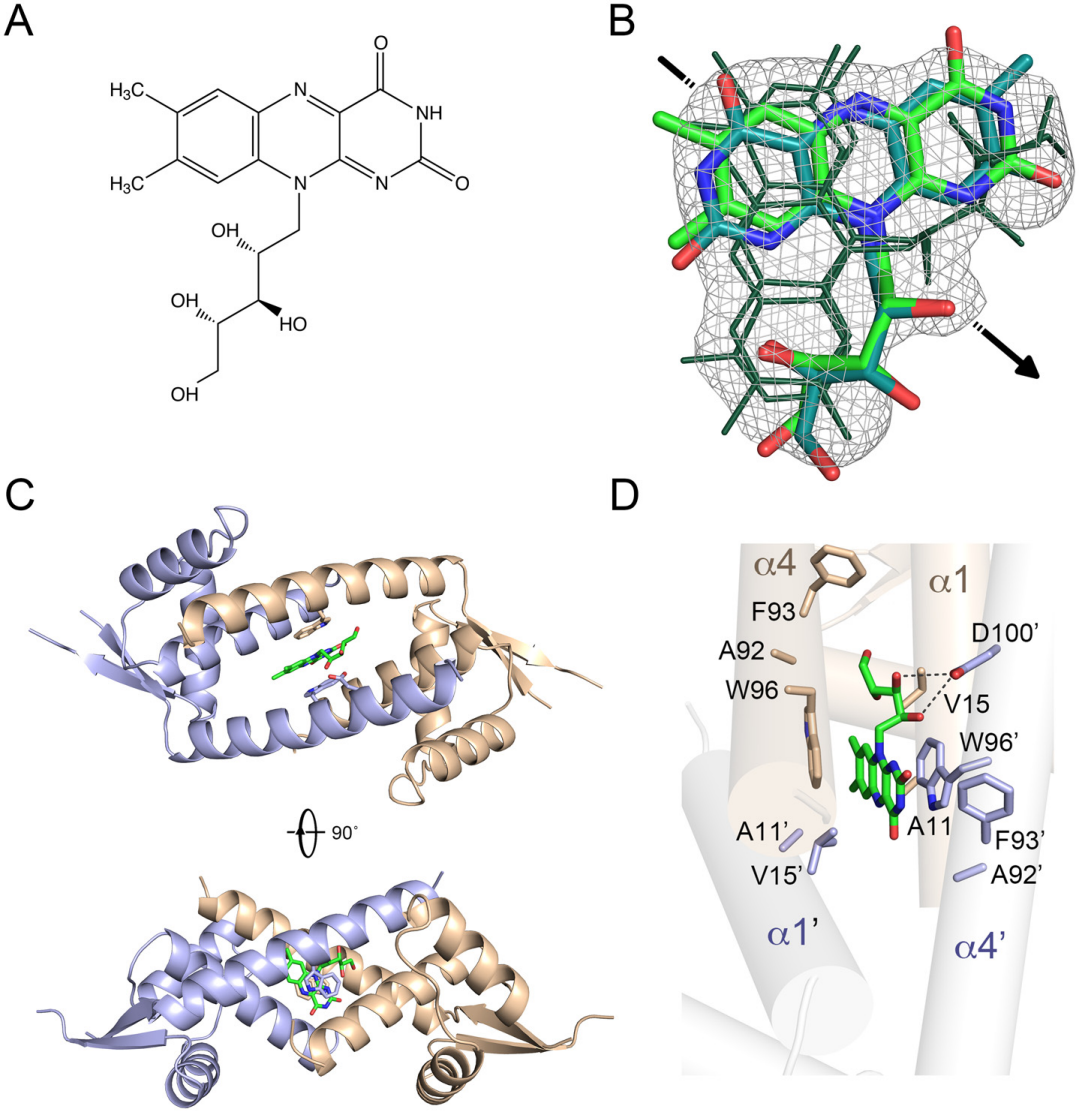
Crystallographic analysis of RBF binding to LmrR. **A**) Chemical structure of RBF. **B**) Composite omit 2*F*_o_-*F*_c_ electron density for RBF in the LmrR•RBF structure calculated at 2.35 Å resolution and contoured at 1σ. The two crystallographically independent binding conformations of RBF are shown in stick representation with the carbon atoms colored green or cyan (oxygen and nitrogen atoms are colored red and blue, respectively). These binding conformations differ by a ~180° rotation of the heterocyclic isoalloxazine core relative to the ribityl moiety. The other two binding modes of RBF (shown with dark green lines) are related to the first two by 2-fold crystallographic symmetry (the location of the crystallographic dyad is indicated with an arrow). C) Overall structure of the LmrR•RBF dimer shown in two orientations. D) Close-up view of the RBF binding site and ligand-interacting residues. Amino acid residues within a radius of 4.5 Å from a ligand are shown in stick representation and labeled.

Riboflavin was identified as a ligand of LmrR by co-crystallization screening. The crystal structure of the riboflavin-bound LmrR complex (LmrR•RBF) was solved by molecular replacement and refined at 2.35 Å (Fig 1, see Table 1 for relevant crystallographic statistics). It has the same crystal form (space group *P*4_3_2_1_2) as the previously determined structure of LmrR bound to H33342 (LmrR•H33342) [11]: a single polypeptide chain occupies the asymmetric unit with the functional dimer being formed by a crystallographic dyad. In the electron density maps, the polypeptide chain is well defined, except for the tip region of the β-wing (residues 70–75) and the N- and C-termini (residues 1–5 and 109–116), which show a high degree of disorder and were therefore excluded from the final model. Extra density in the central drug-binding pore confirmed the presence of bound ligand, but docking of riboflavin was complicated by the occurrence of alternative binding modes (Fig 1B). Different potential binding modes of RBF fitting the electron density were tested by short 200 ps molecular dynamics runs, resulting in the identification of two stable unique binding modes with similar predicted binding energy. These two binding modes of RBF are related by a ~180° flip of the heterocyclic isoalloxazine moiety relative to the ribityl side chain. In addition, each of these two binding modes has a crystallographic symmetry equivalent, due to the symmetrical position of the ligand-binding site in LmrR•RBF on the crystallographic dyad. The occupancy of each of the four binding modes in the crystal was estimated to be 0.25. With the four binding modes superimposed, the isoalloxazine moiety of RBF shows a good fit to the averaged density, but the ribityl side chain is poorly defined, indicating that this moiety is not strongly bound to the protein. Since the four binding modes are highly similar, we will describe only one in detail.

### Binding mode of RBF

**Fig 2:**
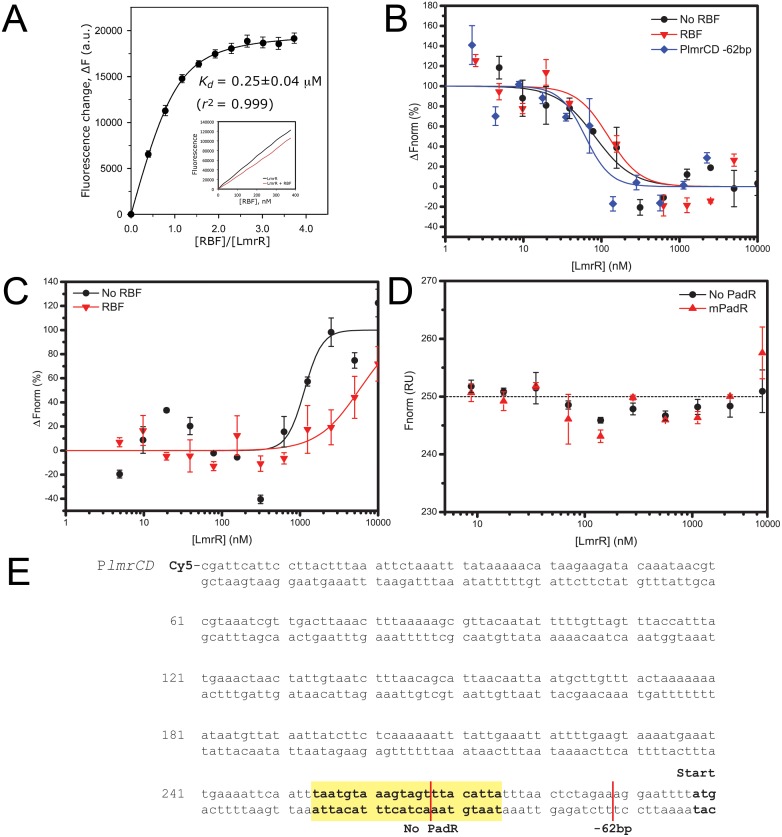
Evaluation of the affinity of LmrR for riboflavin and DNA. A) Fluorescence quenching of riboflavin (RBF) upon titration of LmrR. The fluorescence change was calculated and a binding curve was fitted. Concentration of LmrR in the binding assay was 100 nM (referring to dimeric protein). The inset shows an RBF binding curve obtained with fluorescence quenching data using 1.4 μM LmrR. The indicated standard error of the mean is from two independent experiments. B) Binding of LmrR to P*lmrCD* in the presence (red) and absence (black) of RBF, or to P*lmrCD* shortened by 62 bases (blue) in the absence of RBF. C) Binding of LmrR to P*lmrR* in the presence (red) and absence (black) of RBF. P*lmrR* binding performed at 30% Laser power. D) Binding of LmrR to mutated (mPadR, black squares) and truncated PadR (no PadR, red triangles) and the fitted binding curve (dashed line) that demonstrates a lack of binding. Protein concentrations in panels B-D are for monomeric LmrR. The indicated error bars represent the standard error of the mean with n = 3. E) DNA sequence of the *lmrCD* promoter region, with the palindromic PadR motif shown in bold fond, highlighted in yellow and the transcription start site indicated in bold font. The truncated -62 bp and truncated PadR (No PadR) products are indicated (red lines). A representation of the PadR consensus, the imperfect PadR motif in P*lmrCD* and the mutated P*lmrCD* with the palindromic PadR inverted repeats shown in bold are listed at the bottom.

The overall binding mode of riboflavin is similar to those of H33342 and daunomycin in the previously determined LmrR-ligand structures (Fig 1C and 1D) [11]. The flat aromatic isoalloxazine ring of riboflavin slides into the center of the flat-shaped hydrophobic pore of the LmrR dimer, in between the indole rings of the central Trp96/Trp96’ pair, while the ribityl substituent protrudes towards the pore opening. Riboflavin recognition by LmrR is likely dominated by the aromatic stacking interactions of the Trp96/Trp96’ indole pair with the heterocylic aromatic core of riboflavin. Further stabilization is provided by apolar contacts of the ligand with hydrophobic amino acid residues in helices α1, α4, α1’ and α4’, which face the drug binding pore and surround the central Trp96/Trp96’ pair. Near the pore opening, Asp100 may contribute to the stabilization of the hydrophilic ribityl moiety of riboflavin, by the formation of hydrogen bonds, although the observed disorder of the ribityl moiety indicates that these interactions are quite weak. A similar situation occurs in LmrR•DAU, where Asp100 is found near to the amino sugar substituent of DAU, but does not form strong directive interactions, i.e., hydrogen bonds, as indicated by the high disorder of the substituent [11]. The dissociation constant (K_d_) for binding of RBF to LmrR was determined as 0.26 ± 0.08 μM, using a fluorescence-based titration assay (Fig 2A). A similar Kd was observed at a 14-fold higher LmrR concentration (Fig 2A, inset). It is concluded that the binding affinity of LmrR for riboflavin is similar as for daunomycin (K_d_ = 236 ± 53 nM), but significantly weaker than for H33342 (K_d_ = 21 ± 8 nM) [11].

### Affinity analysis of LmrR to the *lmrR* and *lmrCD promoters*

LmrR has been shown to recognize its specific PadR p/o regions that precede the *lmrR* and *lmrCD*, genes and interact with them in a homodimeric organization [11,12]. However, the data also suggested that LmrR might interact with the two p/o regions in a different way. It was suggested that LmrR binds with two dimers to the *lmrR* p/o region, compared to a single dimer organization on the p/o of *lmrCD* [12]. This tight interaction of LmrR with its promoter causes a strong auto-regulation and repression of the *lmrCD* gene expression but it remained unclear how ligands for LmrR affect the binding. To investigate the importance of the PadR consensus sequence on LmrR-DNA binding several DNA constructs were created containing the wild type and modified *lmrCD* p/o regions (P*lmrCD*) (Fig 2E). Binding of LmrR to the (modified) *lmrCD* promoter region was measured using microscale thermophoresis. The *lmrCD* promoter DNA, synthesized as described in Agustiandari *et al* [12], is strongly bound by LmrR with an apparent K_d_ of 82 ± 7.8 nM (Fig 2B), and a Hill coefficient of 1.78 ± 0.17 which is in agreement with the dimeric assembly of LmrR. Binding of LmrR to the *lmrR* promoter DNA is less strong, with an apparent K_d_ of 1.4 ± 0.1 μM, though with a higher Hill coefficient of 2.43 ± 0.19 (Fig 2C) consistent with the notion that multiple LmrR dimers bind the *lmrR* promoter. The binding curves exhibit an opposite orientation, which we attribute to a probable difference in the LmrR-DNA binding mechanism [12].

Shortening of the 3’ end of the *lmrCD* promoter region by 62 bases slightly improved the LmrR binding, resulting in an apparent K_d_ of 62 ± 7.5 nM (Fig 2B). The 5’ end of the DNA fragment was left unchanged, since this part contains the Cy5 fluorophore and attempts to shorten the fragment from the 5’ end resulted in fluctuations in the fluorescence signal. Mutation of the palindromic PadR motif (Fig 2E) resulted in an abolished LmrR binding (Fig 2D). As a control, half of the PadR consensus was deleted. This DNA fragment did not allow LmrR binding (Fig 2D).

### Riboflavin reduces the affinity of LmrR for its responsive promoters

To investigate the effect of riboflavin on the LmrR-DNA binding we measured the interaction of LmrR with P*lmrCD* and P*lmrR* in the presence of an excess of riboflavin. The use of the ligand riboflavin is advantageous over the other known LmrR ligands, since it does not bind DNA itself, in contrast to H33342 and daunomycin. Addition of riboflavin had a significant effect on the binding of LmrR to its responsive promoter regions (Fig 2), resulting in a shift in apparent K_d_ from 82.1 ± 7.8 nM to 195.4 ± 55.8 nM for P*lmrCD* (Fig 2B) and a shift in apparent K_d_ from 1.4 ± 0.1 μM to 5.12 ± 0.47 μM for P*lmrR* (Fig 2C). Control experiments showed that in the absence of LmrR, riboflavin has no influence on the thermophoretic behavior of these two promoter regions (data not shown).

## Discussion

The PadR consensus is crucial for the binding of LmrR to the *lmrCD* promoter region. Mutagenesis of part of the palindromic motif resulted in an abolished LmrR binding, similar to the removal of half of the PadR consensus sequence. Shortening of the 3’ tail of the DNA fragment improved the binding of LmrR slightly, but this shorter DNA fragment probably diffuses more easily and thereby might improve the dynamics of LmrR binding. Due to the presence of the Cy5 fluorophore on the 5’ end of the DNA fragment and fluctuations in fluorescence signal when modified, the 5’-end was not further shortened.

The crystallographic results and the fluorescence titration assay show that LmrR has the ability to bind riboflavin with an affinity similar as for daunomycin, but significantly weaker than for H33342. The relatively weak interaction is probably due to the fact that riboflavin is not an authentic substrate of LmrCD but that it can bind LmrR because it has a similar structure as the planar heterocyclic LmrR ligands. There is no evidence that LmrCD is involved in detoxification of riboflavin. For instance, high concentrations of riboflavin do not influence the growth of the *lmrCD* deletion strain nor of the wild type *L*. *lactis* (data not shown), and thus shows no particular toxicity. Riboflavin is an essential nutrient for *L*. *lactis* and thus present in used growth media.

Upon addition of an excess of riboflavin to a solution containing LmrR and its DNA binding site a clear drop in binding affinity is observed, i.e., a ~2-fold decrease in LmrR binding to P*lmrCD* and a ~4-fold decrease for P*lmrR*. The usage of the novel LmrR ligand riboflavin has its advantages since riboflavin does not bind DNA, as confirmed by its inability to cause a thermophoresis shift of the promoter DNA in absence of LmrR. A DNA-bound ligand would add molecular mass to the complex and contribute to the thermophoretic diffusion. These data provide direct evidence that binding of LmrR to both the P*lmrCD* and the P*lmrR* is modulated by ligand binding to LmrR. The binding affinity of LmrR for the P*lmrR* is 18-fold poorer than for P*lmrCD*, and this likely allows a basal level of *lmrR* expression. This poorer binding affinity of LmrR for P*lmrR* might be due to differences in the PadR consensus sequence compared to the P*lmrCD* and is consistent with the autoregulatory mechanism of *lmrR* expression, requiring expression also in the absence of drugs.

Previous atomic force microscopy analysis of LmrR-P*lmrR* complexes has shown that P*lmrR* is bound by multiple LmrR dimers [12]. The latter may cause the opposite orientation of the thermophoresis binding curve as compared to P*lmrCD*, that is bound by a single LmrR dimer. These multiple LmrR dimers may bind in a cooperative manner, a hypothesis that is supported by the observation that the hill coefficient for LmrR binding to P*lmrR* is higher (2.43) compared to P*lmrCD* (1.78). We hypothesize that the larger LmrR complex as a whole might not bind P*lmrR* as strongly as the LmrR dimer binds P*lmrCD*, resulting in a poorer K_d_.

**Fig 3:**
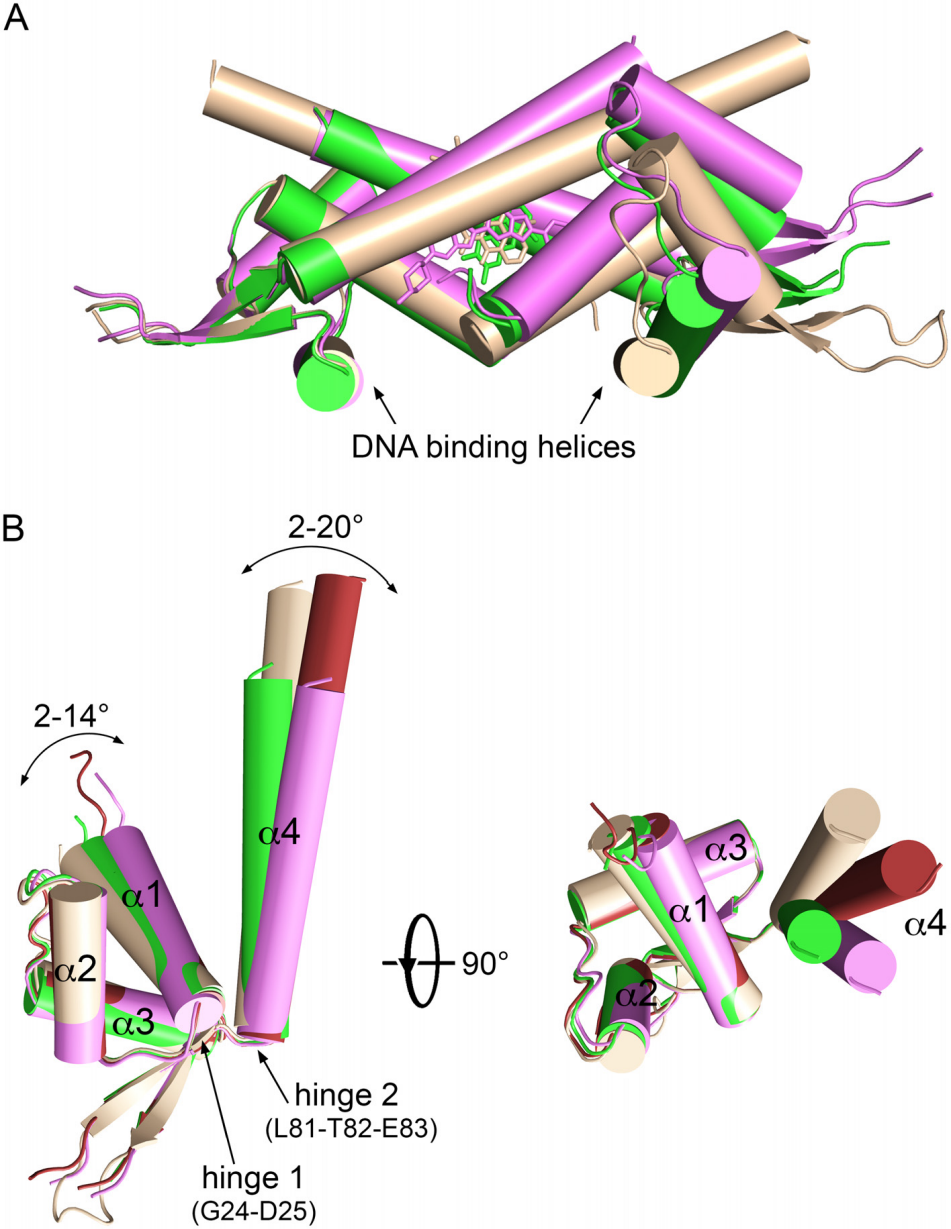
Conformational differences between the three ligand-bound structures of LmrR. A) Superpositions of the three ligand-bound dimers, emphasizing the change in relative disposition of the two DNA-binding helices. B) Superposition of the single subunits of the three ligand-bound LmrR structures in two different views, related by a 90° rotation. For the asymmetric LmrR•DAU dimers both subunits are included in the superposition. The two hinge regions are indicated by arrows, and the ranges by which the orientations of helices α1 and α4 differ are shown. The coloring is as follows: subunit A and B of LmrR•DAU, light and dark brown, subunit A or B of LmrR•RBF, green; subunit A or B of LmrR•H33342, magenta.

Comparison of the LmrR•RBF crystal structure with the previously determined structures of LmrR bound with H33342 and DAU reveals a significant conformational flexibility of the protein, affecting the overall geometries of both the drug and DNA binding sites (Fig 3A). The conformational flexibility largely originates from two hinge regions in the polypeptide chain of LmrR: in the α1-α2 loop and in the loop that connects α4 to the β-wing (Fig 3B). The observed conformational differences support recent findings that multi-drug binding and drug-based induction by LmrR is largely entropy-driven [22]. Binding of a ligand results in a shift of the conformational equilibrium of LmrR towards ensembles, which are incompatible with DNA binding, thus weakening the binding of LmrR to its promoter regions and causing its release.

Our results provide a possible explanation for the regulation of *lmrCD*. In the absence of toxic compounds both P*lmrCD* and P*lmrR* are bound by LmrR dimers, although P*lmrR* is probably occupied by multiple dimers simultaneously. The presence of drugs stimulates the release of LmrR from P*lmrCD*, thereby activating the expression of *lmrCD*. The autoregulation of *lmrR* expression is more complicated, as drugs also induce derepression of *lmrR* expression. Summarizing, our results confirm the importance of the PadR motif for the binding of LmrR to its responsive promoter region P*lmrCD* and show that riboflavin is a ligand of LmrR, which can release LmrR from its promoter regions.
